# Case report: Cushing syndrome clinical presentation related to pigmented nodular adrenal disease

**DOI:** 10.1186/1758-5996-7-S1-A94

**Published:** 2015-11-11

**Authors:** Olga Simone Nebel First, Fernanda Angeli Padula, Amine Wiener Vasconcellos Haddad, Márcio Bousada Franco, Renata Marques, Greta Barriquel Pompermaier

**Affiliations:** 1 Hospital Ipiranga, São Paulo, Brazil

## Background

Patient case report of secondary type2 diabetes description presenting the syndrome of Cushing by PPNAD. SLP, 57, born and living in São Paulo, SP, followed at the Endocrinology Clinic of a Public Hospital, diagnosed with type 2 diabetes mellitus, dyslipidemia, Hypertension and Obesity II, in regular use of medications, presenting inadequate glycemic control and blood pressure. Denied polis or hypoglycemia clinical and referred dietary control. On examination, the patient was hypertensive (BP=140/90mmHg), class II obesity (BMI 34), which is centripetal, dorsal hump, facial plethora and full moon facies. Laboratory tests in September 2014 showed deterioration of the glycemic control (FPG 129 mg/dL and HbA1c 9.1%) and LDL-cholesterol (93mg/dL).

## Purpose

The importance of hypercortisolism research in patients due to inadequate glycemic control.

## Materials and methods

Free Cortisol dosage request in urine 24 h. In return visit, the two urinary free cortisol samples showed increased values (first sample: 717.6 and second sample: 304.5/VR: 30-250mcg/dl). The hypercortisolism was confirmed by non-suppression of serum after 1 mg dexamethasone overnight cortisol 18.6 (Confirmatory is> 1,5mcg/dl). The measurement of serum ACTH showed adrenal etiology (ACTH independent SC). Other pituitary baseline tests and bone densitometry were normal. Abdominal computed tomography and pelvis showed the presence of multiple nodular formations, smaller than 1 cm, grouped with faint contrast enhancement located in the region of above glands.

## Results

The hypothesis being held PPNAD – Primary Pigmented Nodular Adrenal Disease. It was started full insulinization in order to glycemic control, performed pre-operative tests/exams and forwarded to urology team for surgical resolution.

## Conclusion

We observed the importance of hypercortisolism research in this patient due to inadequate glycemic control for the diagnosis of a rare disease, PPNAD. The Primary Pigmented Nodular Adrenal disease is often associated with ACTH independent Cushing's syndrome, characterized by adrenal glands of small or normal size with multiple pigmented and small cortical nodules.

